# Preparation, Modification, and Characterization of Alginate Hydrogel with Nano-/Microfibers: A New Perspective for Tissue Engineering

**DOI:** 10.1155/2013/307602

**Published:** 2013-06-05

**Authors:** Bianca Palma Santana, Fernanda Nedel, Evandro Piva, Rodrigo Varella de Carvalho, Flávio Fernando Demarco, Neftali Lenin Villarreal Carreño

**Affiliations:** ^1^Nucleus of Cellular and Tecidual Biology (NCTBio), Post-Graduate Program in Dentistry, Federal University of Pelotas, Rua Gonçalves Chaves 457, Centro, 96015-560 Pelotas, RS, Brazil; ^2^Department of Operative Dentistry School of Dentistry, Federal University of Pelotas, Rua Gonçalves Chaves 457, Centro, 96015-560 Pelotas, RS, Brazil; ^3^Department of Operative Dentistry School of Dentistry, University North of Paraná (UNOPAR), Rua Marselha, Jardim Piza, 86041-140 Londrina, PR, Brazil; ^4^Technology Development Center, Federal University of Pelotas, Rua Felix da Cunha 809, Centro, 96010-00 Pelotas, RS, Brazil

## Abstract

We aimed to develop an alginate hydrogel (AH) modified with nano-/microfibers of titanium dioxide (nfTD) and hydroxyapatite (nfHY) and evaluated its biological and chemical properties. Nano-/microfibers of nfTD and nfHY were combined with AH, and its chemical properties were evaluated by FTIR spectroscopy, X-ray diffraction, energy dispersive X-Ray analysis, and the cytocompatibility by the WST-1 assay. The results demonstrate that the association of nfTD and nfHY nano-/microfibers to AH did not modified the chemical characteristics of the scaffold and that the association was not cytotoxic. In the first 3 h of culture with NIH/3T3 cells nfHY AH scaffolds showed a slight increase in cell viability when compared to AH alone or associated with nfTD. However, an increase in cell viability was observed in 24 h when nfTD was associated with AH scaffold. In conclusion our study demonstrates that the combination of nfHY and nfTD nano-/microfibers in AH scaffold maintains the chemical characteristics of alginate and that this association is cytocompatible. Additionally the combination of nfHY with AH favored cell viability in a short term, and the addition of nfTD increased cell viability in a long term.

## 1. Introduction

Tissue engineering is a field with potential for designing and constructing tissues or organs to restore their function or even completely replace them. The interchange of responsive cells, morphogens, and scaffolds constitutes the three main elements that grounds tissue engineering [1–6]. Scaffolds are three-dimensional structures used to support and guide the in-growth of cells, forming the template for cell colonization, proliferation as well as being able to provide different sets of physiological signals to the developing tissue [7, 8]. Therefore scaffolds perform the structural and biochemical functions of the native extracellular matrix (ECM) until the cells are able to produce their own ECM [9, 10]. It is well known that the native ECM provides a substrate with specific bioactive molecules that controls cellular process such as cell adhesion, proliferation, migration, differentiation, survival and physical support for cells, characteristics that challenge researchers to elaborate an ideal scaffold [11]. 

The collagen fibers, which the diameter ranges from 50 to 500 nm, are one of the main components of the ECM in tissues that require strength and flexibility (e.g., bone) [10]. Since collagen structure is important for cell attachment, proliferation, and differentiation, nano-/microfibers have been incorporated to different types of scaffold, such as poly(l-lactic acid) (PLLA) [9, 10] and alginate hydrogel [3], in order to recreate collagen fibers functions [12]. Studies have demonstrated that the incorporation of nano-/microfibers in scaffolds can increase osteoblast viability [13], support an earlier and enhanced osteoblast phenotype, increase the expression of genes that are associated with the osteoblast phenotype, and have superior ability to promote mineralization; high expression of integrins *α*2 and *β*1 as well as integrins *α*v and *β*3 and activation of FAKs [10]; nano-/microfibers architecture can selectively enhance protein adsorption (fibronectin and vitronectin) [9]. 

Alginate is a naturally derived polysaccharide that has been widely used in drug delivery [14–16], cell encapsulation material [17], and injectable cell transplantation vehicle [18]. Alginate is composed of (1–4) *β*-d-mannuronic acid and *α*-l-guluronic acid residues linked either randomly or as homopolymeric blocks [19]. The ratio of the two sugars (mannuronic/guluronic acids) is generally 1.5, with some deviation depending on the source [20].

The crosslinking and gelation of the polymers are mainly achieved by the exchange of sodium ions from the guluronic acids with the divalent cations and the stacking of these guluronic groups to form the characteristic egg-box structure [20]. A simple method to increase the ionic crosslinking density is adding sufficient amounts of divalent cations, which is gradually diffuse out from the gel and slowly degrades and is excreted in the urine [18]; in this processes we obtain the proper mechanical properties of alginate hydrogels (AH) [21]. 

Alginate has been frequently used in tissue engineering due to the following properties: biocompatibility, low toxicity, nonimmunogenicity, relatively low cost, and mild gelation behavior with divalent cations [21]. In addition, AH can be used like beads or gel that absorbs water and swell readily without dissolving [22]. In recent years, AH have found applications in medicine [18], pharmacology [15], biological science [23], and dentistry [24] and have been used in large scale in tissue engineering [25]. 

In a previous work we developed a new method to synthesize nano-/microfibers of titanium dioxide (nfTD) and hydroxyapatite (nfHY) and showed that both fibers were not cytotoxic and resembled the structure of natural collagen [3]. In the present study, we developed an alginate hydrogel modified with nano-/microfibers of titanium dioxide (nfTD) and hydroxyapatite (nfHY) and evaluated its biological and chemical properties. 

## 2. Materials and Methods

### 2.1. Alginate Hydrogel Combined with Nano-/Microfibers

Sodium alginate (NaC_6_H_7_O_6_—Vetec Química Fina LTDA) was dissolved in deionized water and mixed with calcium sulfate (CaSO_4_·2H_2_O—Vetec Química Fina LTDA) forming the ionic crosslinking or the hydrogel (2% wt). The nano-/microfibers titanium dioxide and hydroxyapatite kept a constant concentration of 0.07% g/mL; they were added on the hydrogel during the magnetically stirred. 

### 2.2. FTIR Spectroscopy

The chemical structure characterization of alginate hydrogel with and without nano-/micro fibers was conducted by infrared spectroscopy. The infrared spectra of alginate hydrogel were measured with an FTIR spectrophotometer (Fourier Transform Infrared Spectrophotometer, IRPrestige-21, Shimadzu). Each spectrum of samples was acquired via accumulation of 96 scans with a resolution of 4 cm^−1^.

### 2.3. X-Ray Diffraction (XRD)

XRD patterns of dry AH with and without the nano-/microfibers were obtained by diffract meter (XRD, Shimadzu, model XRD-6000). The equipment uses the diffraction tube with copper target at a wavelength approximately equal to 1.54060 Å, with a power of 2 kW, 30 kV current of 30 mA. The analysis was performed in the angle range from 20° to 40° for AH combined with nfTD and from 20° to 80° for AH combined with nfHY, at a speed of 1 degree/min in continuous scan. 

### 2.4. Energy Dispersive X-Ray Analysis (EDX)

The percentage of nfTD and nfHY nano-/microfibers in the AH was determined by Energy Dispersive X-Ray Analysis (EDX—Ray Ny—EDX 720, Shimadzu). Samples were prepared in a similar way to those analyzed by XRD.

### 2.5. Cytocompatibility Test of Alginate Hydrogel Combined with Nano-/Microfibers

An immortalized mouse fibroblast cell line (NIH/3T3) was maintained in Dulbecco's Modified Eagle Medium (DMEM) supplemented with 10% fetal bovine serum (FBS), 2% L-glutamine, penicillin (100 U/mL), and streptomycin (100 mg/mL) (Gibco, Grand Island, NY, USA). Mouse fibroblasts were maintained as a stock culture in DMEM and incubated at 37°C in a humidified atmosphere of 5% CO_2_ in air until subconfluency was reached, as described previously [26, 27]. AH alone and combined with nfTD and nfHY was sterilized by exposure to germicidal UV (ultraviolet) light for 40 min. Subsequently they were incubated in contact with NIH/3T3 cells at a density of 2 × 10^4^ in 96-well plates for 3, 6, and 24 at 37°C in a humidified atmosphere with 5% of CO_2_. An additional control group was added composed only by cell and medium culture, without the presence of AH scaffolds. At each time point, 10 *μ*L of WST-1 (2-(4-iodophenyl)-3-(4-nitrophenyl)-5-(2,4-disulfophenyl)-2H-tetrazolium, monosodium salt) (Roche, Mannheim, Germany) was added to the wells and incubated for 2 h. Then, 100 *μ*L aliquots were removed from each well, and the optical density at 450 nm was determined in a microplate reader. All observations were validated by at least three independent experiments, and for each experiment the analyses were performed in triplicate. Data were submitted to one-way ANOVA and Tukey post-hoc tests, with *P* < 0.05.

## 3. Results

### 3.1. FTIR Spectroscopy Analysis

**Figure 1:**
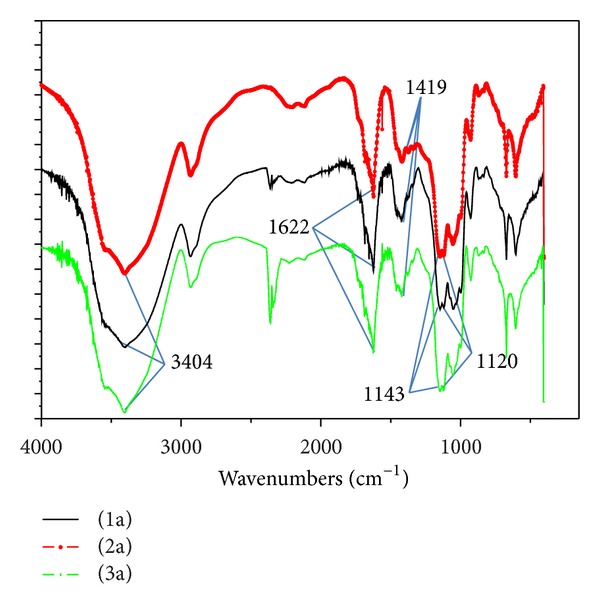
FTIR spectra of HA (1a), AH with nfHY (2a), and AH with nfTD (3a).

Comparing FTIR spectra (Figure 1) of alginate hydrogel (1) with nfHY (2) or nfTD (3), we observe that AH maintained their chemical structure. This can be observed by the characteristic peaks of sodium alginate absorption at 2950 cm^−1^ and 1413 cm^−1^; due to stretching –CH_2_, the carboxylic groups C–O–O show a broad absorption band as a result of the asymmetric stretch in 1622 cm^−1^ and the symmetric stretching in 1419 cm^−1^ and –C–OH (O–H stretching vibration is 3404 cm^−1^, C–O stretching vibration of secondary alcohol is 1120 cm^−1^, and C–O stretching vibration of tertiary alcohol is 1143 cm^−1^).

### 3.2. X-Ray Diffraction (XRD) Analysis

**Figure 2:**
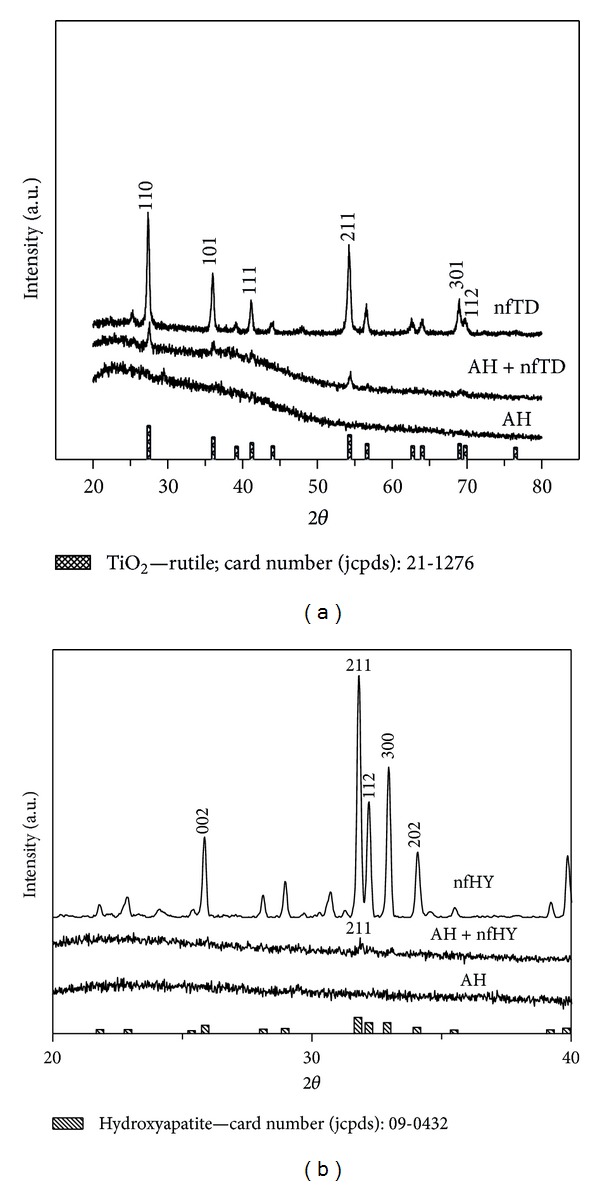
XRD patterns of AH and AH combined with nfTD (a) and nfHY (b).

The presence of titanium dioxide and hydroxyapatite crystal phase in the injectable system was observed by XRD analysis (Figure 2). Results indicated that the nfTD and nfHY preserved thier structural characteristics during the process, which is favorable to maintain its bioactivity and biocompatibility.

### 3.3. EDX

In EDX results we can observe the quantitative concentration of AH (Table 1) combined with nfTD (Table 2) and nfHY (Table 3). 

### 3.4. Cytocompatibility Test of Alginate Hydrogel Combined with Nano/Micro Fibers

**Figure 3:**
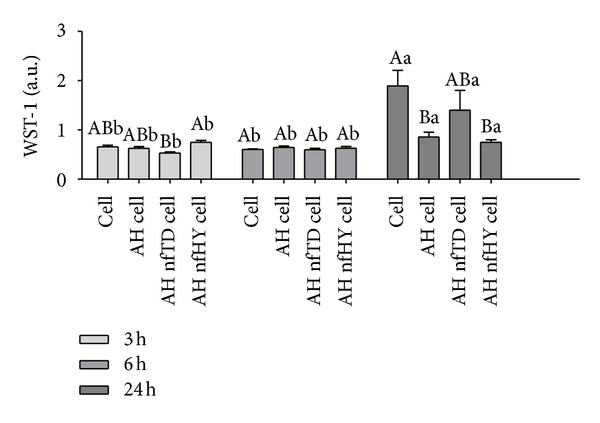
Mouse fibroblast cell line (NIH/3T3) viability in 3, 6, and 24 h when in contact with AH alone and AH combined with nfTD and nfHY. Data are expressed as the mean ± SEM. Uppercase letters indicate significant differences between the AH alone and the AH nfTD and AH nfHY in the same period of time. Lowercase letters indicate significant differences in the times tested. A *P* value < 0.05 was considered significant (Tukey's test).

Cell viability was determined by the WST-1 assay, a soluble tetrazolium salt converted to a deep red colored product by mitochondrial activity [2]. The viability data of NIH/3T3 cells when in contact with AH alone and AH modified with nfTD and nfHY in the period 3, 6, and 24 h are present in Figure 3. 

The results shows that the addition of nfTD and nfHY to the AH scaffold did not induce cytotoxicity. In the period of 24 h the AH nfTD provided a higher viability of NIH/3T3 cells when compared to the AH nfHY and AH alone. However, in the first 3 h AH nfHY showed a slight increase in cell viability when compared to AH alone and associated with nfTD. The exposure time of 3 and 6 h had no significant effect on the cell viability; however, an increase on cell proliferation was observed with 24 h of exposure. 

## 4. Discussion

One well-known limitation of using AH in tissue engineering is the lack of corresponding binding sites for receptors of most cells. Also due to its hydrophilic nature ECM proteins such as laminin, fibronectin, and vitronectin do not readily adsorb to the gel surface [28]. In order to overcome these problems, a common approach has been to combine an entire ECM protein or peptide sequence capable of binding to cellular receptors to the polymer. Combining whole molecules, however, can lead to nonspecific interaction, and the coupling can be difficult to control. Therefore peptide sequences found in the ECM can mediate cell adhesion in place of the larger molecules, offering a specific means to control adhesion and results in a high specificity. The most frequently used is the amino acid sequence arginine-glycine-aspartic acid (Arg-Gly-Asp or RGD) [29]. 

In this study we attempted to modify AH with nfTD and nfHY nano-/microfibers in order to increase cell adhesion. This attempt could favor further improvements of AH properties enhancing cell adhesion and improving tissue formation. The results demonstrate that the association of nfTD and nfHY nano-/microfibers to the AH did not modify the chemical characteristics of the scaffold (Figures 1 and 2). In addition the EDX analysis (Tables 1, 2, and 3) showed that the concentration evaluated was favorable to maintain the original properties of AH.

The cytocompatibility assay showed that the addition of nfTD and nfHY to the AH scaffold did not induce cytotoxicity. These results are in agreement with our recent publication, where an *in vitro* cytocompatibility assay demonstrated that the same nano-/micro fibers alone were not cytotoxic to NIH/3T3 cells [3]. In the first 3 h of culture with NIH/3T3 cells AH nfHY showed a slight increase in cell viability when compared to AH alone and associated with nfTD. This could be partially explained by the higher porosity shown by the alginate with nfHY in contrast with the alginate with nfTD and AH alone, favoring cell adhesion, proliferation, and migration which could improve initially the cell viability [3]. However, an increase in cell viability was observed in 24 h when nfTD was associated with AH scaffold, which could be partially explained by the flowing characteristics of titanium and nanofibrous.

Titanium has been classified as a cytocompatible material, and it has been extensively used in dentistry [30] and orthopedics [31, 32]. It is capable of forming an active oxide layer that readily interacts with cell-surface proteins and with the ECM proteins produced by cells. It is due to this superficial oxide that titanium provides a biocompatibility interface with peri-implant tissue [33]. It has been shown that when, mesenchymal stem cells are cultured with titanium fragments the cell viability improves, and their biology properties are maintained [34]. Further *in vitro* studies have demonstrated that titanium dioxide scaffold can provide a suitable surface for osteoblast cell attachment and proliferation [35]. 

In addition, it is well established that in order to proliferate, migrate and differentiate most cells require anchorage. Therefore cellular attachment is an essential step towards developing a new tissue. It is believed that the adhesion of cells to surfaces is dependent on the adsorption of highly adhesive proteins that can be from the serum or secreted by the cells, which links cells to the biomaterial surface [9, 31]. In this context several key attachment proteins (fibronectin, vitronectin, and laminin) have been found to adsorb to the nanofibrous scaffolds at levels of 2.6 to 3.9 times higher than solid-walled scaffolds. In addition it has been shown that nanofibrous scaffolds adsorb a different profile of proteins in comparison to solid-walled scaffolds from the same material [9]. Nanofibrous scaffolds also have shown to increase in neonatal mouse osteoblasts the expression of integrins associated with collagen (*α*2*β*1), fibronectin (*α*V*β*3), and vitronectin (*α*V*β*3) when compared with solid-walled scaffolds [10]. In addition nanofibrous scaffolds have shown to increase cell attachment with several cell lines including osteoblastic cells [13], fibroblasts, normal rat kidney cells [36, 37], smooth muscle cells [38], and neural stem cells [39]. 

## 5. Conclusion

In our study we demonstrated that the combination of nfHY and nfTD nano-/microfibers in alginate hydrogel scaffold maintains the chemical characteristics of alginate, and that this association is cytocompatible. Additionally the combination of nfHY with AH favored cell viability in a short term, and the addition of nfTD increased cell viability in a long term.

## Figures and Tables

**Table 1:** Quantitative analyses of alginate hydrogel.

Sample	Results	SD	Line analysis of X-ray
Ca	77.09%	0.23	Ca Ka
S	20.76%	0.10	S Ka
P	1.21%	0.06	P Ka
Fe	0.57%	0.04	Fe Ka
Cu	0.37%	0.02	Cu Ka

**Table 2:** Quantitative analyses of alginate hydrogel combined with nano-/microfibers of titanium dioxide.

Sample	Results	SD	Line analysis of X-ray
Ca	62.31%	0.11	Ca Ka
S	26.05%	0.05	S Ka
Ti	10.44%	0.03	Ti Ka
P	1.03%	0.02	P Ka
K	0.17%	0.01	K Ka

**Table 3:** Quantitative analyses of alginate hydrogel combined with nano-/microfibers of hydroxyapatite.

Sample	Results	SD	Line analysis of X-ray
Ca	77.55%	0.07	Ca Ka
S	11.31%	0.02	S Ka
P	10.44%	0.02	P Ka
Si	0.35%	0.01	Si Ka
K	0.25%	0.01	K Ka
Fe	0.11%	0.00	Fe Ka
